# Effectiveness of antiretroviral therapy in the single-tablet regimen era

**DOI:** 10.11606/S1518-8787.2018052000399

**Published:** 2018-11-14

**Authors:** Juliana de Oliveira Costa, Maria das Graças Braga Ceccato, Micheline Rosa Silveira, Palmira de Fátima Bonolo, Edna Afonso Reis, Francisco de Assis Acurcio

**Affiliations:** I Universidade Federal de Minas Gerais. Faculdade de Medicina. Programa de Pós-Graduação em Saúde Pública. Belo Horizonte, MG, Brasil; II Universidade Federal de Minas Gerais. Faculdade de Farmácia. Departamento de Farmácia Social. Belo Horizonte, MG, Brasil; III Universidade Federal de Minas Gerais. Faculdade de Medicina. Departamento de Medicina Preventiva e Social. Belo Horizonte, MG, Brasil; IV Universidade Federal de Minas Gerais. Instituto de Ciências Exatas. Departamento de Estatística. Belo Horizonte, MG, Brasil

**Keywords:** Anti-HIV Agents, administration & dosage, Antiretroviral Therapy, Highly Active, Evaluation of the Efficacy-Effectiveness of Interventions, Cohort Studies, Fármacos Anti-HIV, administração & dosage, Terapia Antirretroviral de Alta Atividade, Avaliação de Eficácia-Efetividade de Intervenções, Estudos de Coortes

## Abstract

**OBJECTIVE:**

To evaluate the effectiveness of antiretroviral therapy and the associated factors according to the type of regimen used: Single Tablet Regimen or Multiple Tablet Regimen.

**METHODS:**

Prospective cohort of 440 patients (male, 74.3%, median age, 36 years old) who initiated antiretroviral therapy between Jan/14 and Dec/15 at a referral service in Belo Horizonte. Efficacy was defined as viral suppression (viral load, VL < 50 copies/ml) and evaluated after six and twelve months of treatment. Sociodemographic, clinical and behavioral data were collected from clinical charts and from Information Systems. Multivariate analysis of overall effectiveness was performed by logistic regression.

**RESULTS:**

Most patients initiated Multiple Tablet Regimen antiretroviral therapy (n = 255, 58%). At six months, overall viral suppression was 74.6%, being higher among patients who used Single Tablet Regimen (80.6%, p = 0.04). At twelve months, 83.2% of patients reached viral suppression, with no difference between groups (p = 0.93). Factors independently associated with viral suppression at six and twelve months varied, being negatively associated with effectiveness: VL ≥ 100,000 copies/ml, symptoms of AIDS, longer interval time between diagnosis and initiation of antiretroviral therapy, antiretroviral switching, smoking or current illicit drugs usage (p < 0.05). Factors positively associated with viral suppression included adherence to antiretroviral therapy and category of risk/exposure of men who have sex with men (p < 0.05). Reaching viral suppression at six months was the main predictor of effectiveness at one year (OR = 8.96 and p < 0.01).

**CONCLUSIONS:**

Viral suppression was high and better results were achieved for patients who used Single Tablet Regimen regimens at six months. Clinical, behavioral, and antiretroviral therapy -related factors influence viral suppression and highlight the need for interventions to increase early diagnosis and initiation of antiretroviral therapy, patient’s adherence, and to reduce illicit drugs and cigarette smoking in this population.

## RESUMO

**OBJETIVO:**

Avaliar a efetividade da terapia antirretroviral e fatores associados segundo o tipo de esquema utilizado: medicamento em dose fixa combinada ou múltiplos medicamentos e doses.

**MÉTODOS:**

Coorte prospectiva não concorrente de 440 pacientes que iniciaram terapia antirretroviral entre janeiro de 2014 e dezembro de 2015 em Belo Horizonte, MG. A efetividade foi definida como supressão viral (carga viral [CV] < 50 cópias/ml) e avaliada após seis e 12 meses de tratamento. Dados sociodemográficos, clínicos e comportamentais foram coletados de prontuário clínico e de sistemas de informação. A análise múltipla da efetividade global foi realizada por regressão logística.

**RESULTADOS:**

A maioria dos pacientes iniciou terapia antirretroviral com múltiplos medicamentos e doses (58%). Aos seis meses, a supressão viral global foi 74,6%, maior entre pacientes que utilizaram dose fixa combinada (80,6%; p = 0,04). Aos 12 meses, 83,2% dos pacientes atingiram supressão viral, sem diferença entre os grupos (p = 0,93). Fatores independentemente associados à supressão viral em seis e 12 meses variaram, e foram negativamente associados à efetividade: CV ≥ 100.000 cópias/ml, sintomas definidores de aids, maior intervalo de tempo entre diagnóstico e início da terapia antirretroviral, troca de antirretroviral e consumo de tabaco ou drogas ilícitas (p < 0,05). Fatores positivamente associados à supressão viral incluíram adesão à terapia antirretroviral e categoria de risco/exposição de homens que fazem sexo com homens (p < 0,05). Atingir supressão viral aos seis meses foi o principal preditor de efetividade em um ano (OR = 8,96; p < 0,01).

**CONCLUSÕES:**

A supressão viral foi elevada e superior para pacientes que utilizaram esquemas de dose fixa combinada aos seis meses. Fatores clínicos, comportamentais e relacionados à terapia antirretroviral influenciaram a supressão viral e evidenciam a necessidade de intervenções para aumentar o diagnóstico, o início precoce e a adesão dos pacientes à terapia antirretroviral, bem como reduzir o uso de drogas ilícitas e tabaco nesta população.

## INTRODUCTION

An estimated 830,000 people were living with HIV in Brazil by the end of 2016, a prevalence of 0.4% in the general population. This prevalence is higher among sex workers, injecting drug users, men who have sex with men (MSM), and persons deprived of liberty^a^ ^,^ ^b^. Of this total, about 498 thousand people use antiretroviral therapy (ART), a coverage rate of 60%^b^.

Since 1996, the Brazilian Unified Health System (SUS) has offered ART as part of its care policy for people living with HIV (PLHIV)^1^. Currently, 22 antiretroviral drugs (ARV) are provided for HIV control, including the single-tablet regimen (STR) of tenofovir, lamivudine and efavirenz, listed in 2015^c^.

The STR have allowed the simplification of ART and the administration of a single tablet per day compared to multiple-tablet regimens (MTR). The benefits include greater patient preference, increased self-perceived health, greater adherence to ART, greater viral suppression, better laboratory parameters and reduction of associated costs^2^. Thus, several clinical protocols, in agreement with the recommendations of the World Health Organization, favor the use STR as initial therapy^2^ ^,^ ^d^.

The Brazilian Ministry of Health invested over R$ 1 billion in antiretroviral drugs and the treatment of sexually transmitted diseases in 2016^e^. Despite the high investment, studies on the effectiveness of these drugs in Brazil are scarce. Recently published studies^3^ ^,^ ^4^ are restricted to patients who initiated ART by 2010, when STR were not available and recommendations for initial ART depended on CD4 + T lymphocytes (CD4 + TL) and viral load (VL) levels of patients, as opposed to the current recommendation to initiate treatment promptly, regardless of such parameters^c^. Added to that is the lack of observational studies in ART-naïve patients who used specifically the STR containing tenofovir, lamivudine and efavirenz.

We aimed to evaluate the effectiveness of antiretroviral drugs in PLHIV according to the type of regimen (STR or MTR) used in the treatment and associated factors.

## METHODS

A non-concurrent prospective cohort of patients who initiated ART between January 2014 and December 2015 at an HIV specialized service (SAE) that provides inpatient and outpatient care in Belo Horizonte, the major point of reference in Minas Gerais.

A total of 1,249 patients were identified in *Sistema de Controle Logístico de Medicamentos* (Siclom – Logistic Control System of Medicines) and in HIV and AIDS reports issued by the SAE. Of those, 440 met the inclusion criteria (Figure). The following were considered eligible: patients aged 16 or over, diagnosed with HIV, ART-naïve and with at least one outpatient healthcare visit after initiating ART. Pregnant women were excluded due to different therapy indications.

**Figure:**
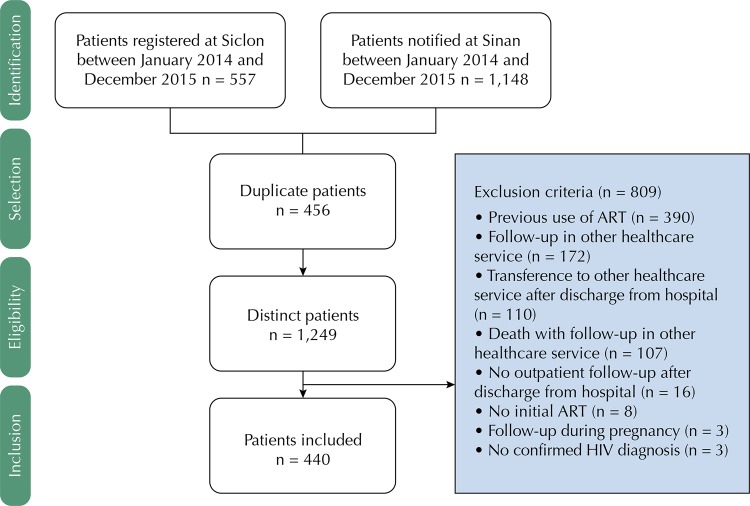
Diagram of patients’ inclusion in the cohort. Belo Horizonte, State of Minas Gerais, 2015.

Siclom: *Sistema de Controle Logístico de Medicamentos* (Logistic Control System of Medicines); Sinan: *Sistema de Informação de Agravos de Notificação* (Information System for Notifiable Diseases); ART: Antiretroviral therapy

The data were collected from medical records, Siclom and *Sistema de Controle de Exames Laboratoriais da Rede Nacional de Contagem de Linfócitos CD4+/CD8+ e Carga Viral* (Siscel – Laboratory Examination Control System) The data collected in medical records were: sociodemographic (sex, race, age, marital status, level of education, children, employment); behavioral and lifestyle habits (use of tobacco, alcohol and illicit drugs, prior and during follow-up); clinical/laboratory [possible sources of infection, hospitalizations in the previous year and after initiating ART, initial clinical classification of patient (according to adapted criteria of the Centers for Disease Control and Prevention^f^) and diagnoses of comorbidities and coinfections (according to criteria of the International Classification of Diseases, tenth revision^g^)]; and drug therapy (time between diagnosis and initial ART; therapy regimen used; record of adverse events; and adherence, characterized by the absence of non-adherence notes in the medical record).

The ARV switch was collected from Siclom and defined as replacement of an initially prescribed active ingredient. The results of the CD4 + TL and VL counts performed during follow-up were collected from Siscel or, when unavailable, from the patient’s medical record.

Data collection was carried out by two trained researchers using a standardized form and typed in EpiInfo^®^ 3.5.4 software. Collection and typing quality was verified by recollecting and retyping 10% of medical records with Kappa statistic value, indicating perfect inter-examiner agreement (k = 0.92)^5^.

Patient follow-up lasted 12 months. The initial date of therapy was determined by the first dispensation of ART to outpatients registered with Siclom or the first dispensation of ART registered in the medical record of patients who began therapy during hospitalization.

Therapy effectiveness was defined as viral suppression (plasma VL < 50 copies/ml) six months after initial treatment^c^. Immunological effectiveness and recovery were evaluated as secondary outcomes (increase of more than 30% of CD4 + levels^c^) after 12 months of follow-up. A three-month tolerance was used to collect test results to reduce the amount of missing data.

Patients were compared according to initial therapy regimen: STR or MTR. Categorical variables were presented by frequency distribution and quantitative variables by central tendency (mean or median) and variability (SD: standard deviation and TA: total amplitude). The chi-square test was used to compare the groups.

The magnitude of the association between the explanatory variables and effectiveness was estimated by odds ratio with 95% confidence interval. The independent effect of the explanatory variables was evaluated by a multiple logistic regression model which included all explanatory variables that obtained p < 0.20 in the Wald test in simple regression. The goodness of fit of the multiple model was verified by the area under the Receiver Operating Characteristic curve (above 0.7). The level of significance was 5% for all analyses, performed using R software version 3.4.0.

Three scenarios were constructed to evaluate the impact of missing data on the analysis outcomes: in the first, only patients with observed data were evaluated; in the second, missing data were considered as success (VL < 50 copies/ml), and, in the third, missing data were considered as failure (VL ≥ 50 copies/ml).

The missing data were replaced using multiple imputation by chained equations (MICE package, m = 20)^6^. The percentage of missing data was up to 30% for explanatory variables, and 23.8% at six months and 22.7% at 12 months for effectiveness. Imputation consistency was assessed by Pearson correlation between the predicted values of the estimated model using only observed data and data of the imputed model (R^2^ = 0.99 for six months and R^2^ = 0.83 for 12 months). No differential loss was observed between the groups that reached viral suppression or not according to the explanatory variables.

This study is part of the ECOART project *“Efetividade da terapia antirretroviral em pessoas vivendo com HIV, HIV/tuberculose, HIV/hanseníase ou HIV/leishmaniose visceral, acompanhados em Belo Horizonte”* (Effectiveness of antiretroviral therapy in people living with HIV, HIV/tuberculosis, HIV/leprosy or HIV/visceral leishmaniasis, followed up in Belo Horizonte) approved by the Research Ethics Committee of Universidade Federal de Minas Gerais (CAAE 31192014.3.0000.5149; 2014) and Hospital Eduardo de Menezes (877,392).

## RESULTS

The median follow-up time was 11 months; 22.0% of patients were hospitalized and 11 died over this period. Most patients were male (74.3%) and the main category of risk or exposure to HIV infection was MSM (32.3%), followed by heterosexual men (30.0%). Many patients entered the cohort with advanced immunosuppression, characterized by CD4 + counts below 200 cells/mm^3^ (32.0%) and presence of AIDS-defining signs and symptoms (45.7%), although most had no record of hospitalization due to HIV in the year prior to entering the cohort (52.7%) (Table 1).

**Table 1:** Characteristics of patients included in the study. Belo Horizonte, State of Minas Gerais, 2015. (n = 440)

Variable	Total (n = 440)		STR (n = 185)		MTR (n = 255)	
		
	n	%	n	%	n	%
Sociodemographic						

Sex						
Female	113	25.7	43	23.2	70	27.5
Male	327	74.3	142	76.8	185	72.5
Age (years)						
16 to 36	218	49.5	91	49.2	127	49.8
> 36 to 77	222	50.5	94	50.8	128	50.2
Level of education						
Secondary or higher education	194	44.1	97	52.4^a^	97	38.0
Illiterate to complete primary education	175	39.8	65	35.1	110	43.1
Missing data	71	16.1	23	12.4	48	18.8
Race/skin color						
Non-brown	163	37.0	84	45.4^a^	79	31.0
Brown	272	61.8	99	53.5	173	67.8
Missing data	5	1.1	2	1.1	3	1.2
Marital status						
Married/Stable union	128	29.1	54	29.2	74	29.0
Divorced/Single/Widowed	310	70.5	130	70.3	180	70.6
Missing data	2	0.5	1	0.5	1	0.4
Employment						
Yes	224	50.9	90	48.6	134	52.5
No	182	41.4	76	41.1	106	41.6
Missing data	34	7.7	19	10.3	15	5.9
Children						
Yes	204	46.4	86	46.5	118	46.3
No	190	43.2	76	41.1	114	44.7
Missing data	46	10.5	23	12.4	23	9.0

Clinical/Laboratory						

Risk/Exposure category						
Heterosexual female	100	22.7	36	19.5	64	25.1
Heterosexual male	132	30.0	56	30.3	76	29.8
MSM	142	32.3	57	30.8	85	33.3
Injecting drugs/Other	13	3.0	6	3.2	7	2.7
Missing data	53	12.0	30	16.2	23	9.0
Viral load before ART						
Up to 100,000 copies/ml	179	40.7	88	47.6	91	35.7
> 100,000 copies/ml	129	29.3	51	27.6	78	30.6
Missing data	132	30.0	46	24.9	86	33.7
CD4+ before ART						
> 500 cells/mm^3^	77	17.5	33	17.8	44	17.3
201 to 499 cells/mm^3^	98	22.3	37	20.0	61	23.9
Up to 200 cells/mm^3^	141	32.0	65	35.1	76	29.8
Missing data	124	28.2	50	27.0	74	29.0
Medical condition^c^						
With AIDS (C)	201	45.7	82	44.3	119	46.7
Without AIDS (A/B)	239	54.3	103	55.7	136	53.3
Hospitalizations in previous year						
0	232	52.7	104	56.2	128	50.2
1	156	35.5	56	30.3	100	39.2
2 or more	52	11.8	25	13.5	27	10.6
Hospitalizations during follow-up						
0	343	78	147	79.5	196	76.9
1	77	17.5	29	15.7	48	18.8
2 or more	20	4.5	9	4.9	11	4.3
Hepatitis B or C during follow-up						
Yes	10	2.3	6	3.2	4	1.6
No	430	97.7	179	96.8	251	98.4
Mental disorder during follow-up						
Yes	114	25.9	34	18.4^a^	80	31.4
No	326	74.1	151	81.6	175	68.6

ART-related						

Initial treatment						
Outpatient unit	280	63.6	126	68.1	154	60.4
Hospital	160	36.4	59	31.9	101	39.6
Year						
2014	210	47.7	-	-	210	82.4
2015	230	52.3	185	100	45	17.6
Initial ART regimen (2 NRTI)						
INI	1	0.2	-	-	1	0.4
PI	34	7.7	-	-	34	13.3
NNRTI	405	92	185	100	220	86.3
Time between diagnosis and ART						
Up to 2 months	231	52.5	108	58.4^a^	123	48.2
> 2 months	209	47.5	77	41.6	132	51.8
Record of AR to ART during follow-up						
Yes	224	50.9	65	35.1^a^	159	62.4
No	216	49.1	120	64.9	96	37.6
ARV switch^b^ during follow-up						
Yes	105	23.9	28	15.1^a^	77	30.2
No	335	76.1	157	84.9	178	69.8
Record of adherence to ART in 6 months						
Yes	367	83.4	158	85.4	209	82
No	73	16.6	27	14.6	46	18
Record of adherence to ART in 12 months						
Yes	323	73.4	140	75.7	183	71.8
No	117	26.6	45	24.3	72	28.2

Behavioral and lifestyle						

Previous tobacco use						
Yes	223	50.7	98	53.0	125	49.0
No	132	30.0	61	33.0	71	27.8
Missing data	85	19.3	26	14.1	59	23.1
Previous alcohol use						
Yes	288	65.5	136	73.5	152	59.6
No	67	15.2	28	15.1	39	15.3
Missing data	85	19.3	21	11.4	64	25.1
Previous illicit drugs use						
Yes	142	32.3	76	41.1	66	25.9
No	179	40.7	82	44.3	97	38.0
Missing data	119	27.0	27	14.6	92	36.1
Tobacco use during follow-up						
Yes	148	33.6	59	31.9	89	34.9
No	264	60.0	114	61.6	150	58.8
Missing data	28	6.4	12	6.5	16	6.3
Alcohol use during follow-up						
Yes	202	45.9	90	48.6	112	43.9
No	204	46.4	81	43.8	123	48.2
Missing data	34	7.7	14	7.6	20	7.8
Illicit drugs use during follow-up						
Yes	71	16.1	35	18.9	36	14.1
No	302	68.6	123	66.5	179	70.2
Missing data	67	15.2	27	14.6	40	15.7

STR: single-tablet regimen; CD4+: CD4+T lymphocites; MSM: men who have sex with men; MTR: multiple-tablet regimen; INI: integrase inhibitors; PI: protease inhibitors; NRTI: nucleoside/nucleotide reverse transcriptase inhibitors; NNRTI: non-nucleoside reverse transcriptase inhibitor; AR: adverse reaction; ARV: antiretroviral drug; ART: antiretroviral therapy

^a^ p < 0.05 in the chi-square test for observed data.

^b^ Replacement of an initially prescribed active ingredient by another.

^c^ Clinical classification according to adapted criteria of Centers for Disease Control and Prevention; A: asymptomatic, B: symptomatic, C: AIDS-defining symptoms.

During follow-up, 60.7% of patients recorded infectious and parasitic diseases, of whom 28.9% presented HIV complications and 18.2% infections with a predominantly sexual mode of transmission. Co-infection by tuberculosis and hepatitis B or C occurred in 10.0% and 2.3% of the population, respectively. In addition, 25.9% of patients had a record of mental or behavioral disorder, the most common being depression (n = 67, 15.2%) and anxiety (n = 22, 5.0%).

The ART was mainly initiated at outpatient level (63.6%) with regimens that combined two nucleoside/nucleotide reverse transcriptase inhibitors (NRTI) associated with a non-nucleoside reverse transcriptase inhibitor (NNRTI) (92.0%) – mainly efavirenz (90.7%). The most common drug combination was tenofovir, lamivudine and efavirenz (85.0%), followed by zidovudine, lamivudine and efavirenz (3.6%). The interval between diagnosis and initial ART was up to 60 days for approximately half of patients (52.5%), with an average of 1.1 years (SD = 2.7; TA = 21). Adherence to treatment was higher in the first six months, 83.4% of patients, versus 73.4% at 12 months. At least one incident ART-related adverse reaction was recorded for 50.9% of patients and ARV switch for 23.9%. The use of illicit drugs and tobacco reduced after initial ART (Table 1).

The distribution of patients in the STR and MTR groups was mostly similar regarding sociodemographic, clinical, ART-related and behavioral and lifestyle characteristics. However, compared to the MTR group, patients who used STR had a higher level of education, fewer were browns, had a lower prevalence of mental disorders, more recent HIV diagnosis, and lower incidence of adverse reactions to ART and ARV switch (p < 0.05) (Table 1).

Viral suppression was observed for 74.6% of patients at six months of follow-up, and this percentage was higher among patients in the STR group (p = 0.035). Viral suppression results in the other scenarios followed the same trend, although statistical significance was observed only in the success scenario (Table 2).

**Table 2:** Effectivity results according to follow-up time and evaluated scenario. Belo Horizonte, State of Minas Gerais, 2015. (n = 440)

Effectiveness rate	Overall (n = 440)		STR (n = 185)		MTR (n = 255)	
		
	n	%	n	%	n	%
6 months						

Viral load						
Observed data	250/335	74.6	112/139	80.6*	138/196	70.4
Success scenario	355/440	80.7	158/185	85.4*	197/255	77.3
Failure scenario	250/440	56.8	112/185	60.5	138/255	54.1

12 months						

Viral load						
Observed data	283/340	83.2	116/139	83.5	167/201	83.1
Success scenario	383/440	87.0	162/185	87.6	221/255	86.7
Failure scenario	283/440	64.3	116/185	62.7	167/255	65.5
Immunological recovery						
Observed data	155/206	75.2	54/67	80.6	101/139	72.7
Success scenario	389/440	88.4	172/185	93.0*	217/255	85.1
Failure scenario	155/440	35.2	54/185	29.2*	101/255	39.6

STR: single-tablet regimen; MTR: multiple-tablet regimen

* p < 0.05 in the chi-square test.

Observed data: patients who presented observed data; Success scenario: all missing data are considered success; Failure scenario: all missing data are considered failure.

At 12 months of follow-up, 83.2% of the patients reached viral suppression, with no statistically significant difference between the STR and MTR groups (p = 0.929) in all proposed scenarios. In this period, 75.2% of patients reached immunological recovery, also without differences between the groups. However, the percentage of missing data for this outcome was high (53.2%), which may compromise the interpretation of results in the proposed scenarios (Table 2).

Bivariate analysis with imputed data showed different factors associated with viral suppression at six and 12 months. Sociodemographic characteristics did not influence the outcome, whereas clinical variables related to disease progression and immunosuppression at the baseline were associated with lower effectiveness: presence of AIDS-defining signs and symptoms and hospitalization records in the year prior to entry into the cohort and viral load above of 100,000 copies/ml for effectiveness at six and 12 months (p < 0.05) (Table 3).

**Table 3:** Bivariate analysis of effectiveness evaluated by viral suppression according to follow-up time and patients’ characteristics. Belo Horizonte, State of Minas Gerais, 2015. (n = 440)

Variable	6 months			12 months		
	
	OR	95%CI	p	OR	95%CI	p
Sociodemographic						

Sex (male)	0.62	0.34–1.12	0.115	0.61	0.30–1.18	0.159
Age (> 36 years)	0.83	0.51–1.37	0.474	1.33	0.75–2.36	0.333
Level of education (up to complete primary)	0.78	0.48–1.27	0.314	0.97	0.56–1.67	0.900
Race/Skin color (brown)	0.62	0.38–1.02	0.060	1.10	0.60–2.00	0.763
Marital status (single, divorced, widowed)	1.03	0.62–1.71	0.912	1.26	0.69–2.31	0.451
Employment (no)	1.23	0.72–2.08	0.445	1.46	0.81–2.64	0.203
Children (yes)	0.88	0.53–1.47	0.626	0.71	0.40–1.27	0.245

Clinical						

HIV Risk/Exposure (MSM *versus* heterosexuals, IDU, others)	1.00	0.58–1.72	0.992	1.59	0.80–3.16	0.183
Viral load before ART (> 100,000 copies/ml)	0.35	0.19–0.65	0.001	0.54	0.32–0.94	0.030
AIDS^a^ (yes)	0.27	0.16–0.46	0.000	0.57	0.32–1.01	0.053
Hepatitis B or C (yes)	0.50	0.14–1.81	0.289	-	-	0.999
Mental disorder (yes)	0.63	0.37–1.10	0.101	0.61	0.33–1.12	0.108
Hospitalization in previous year (yes)	0.45	0.27–0.75	0.002	0.87	0.49–1.53	0.625
Hospitalization during follow-up (yes)	0.72	0.40–1.27	0.255	1.01	0.49–2.07	0.981

ART-related						

Initial treatment (hospital level)	0.40	0.24–0.66	0.000	0.91	0.50–1.64	0.749
Initial year of treatment (2015)	1.73	1.05–2.85	0.030	1.00	0.57–1.77	0.999
Initial ART regimen (PI *versus* NNRTI)	0.70	0.29–1.69	0.431	0.68	0.26–1.77	0.431
Initial ART regimen (MTR)	0.57	0.34–0.97	0.036	0.97	0.55–1.74	0.929
Time between diagnosis and ART (> 2 months)	1.70	1.02–2.84	0.043	0.68	0.39–1.21	0.189
Record of AR to ART (yes)	0.88	0.53–1.43	0.597	0.99	0.56–1.74	0.963
ARV switch^b^ (yes)	0.24	0.14–0.42	0.000	0.52	0.28–0.95	0.035
Adherence to ART in 6 months (yes)	2.28	1.25–4.14	0.007	-	-	-
Adherence to ART in 12 months (yes)	-	-	-	4.04	2.24–7.31	0.000
Effectiveness at 6 months (yes)	-	-	-	7.78	3.83–15.78	0.000

Behavioral and lifestyle						

Tobacco use in life (yes)	0.72	0.41–1.28	0.265	0.60	0.31–1.16	0.127
Alcohol use in life (yes)	0.86	0.46–1.28	0.631	0.76	0.35–1.67	0.487
Drug use in life (yes)	0.66	0.40–1.06	0.087	0.52	0.27–1.01	0.053
Recent tobacco use (sim)	0.49	0.31–0.78	0.003	0.43	0.23–0.80	0.009
Alcohol use during follow-up (yes)	1.00	0.58–1.71	0.991	0.75	0.41–1.36	0.335
Drug use during follow-up (yes)	0.66	0.34–1.28	0.222	0.39	0.21–0.73	0.004

CD4+: CD4+T lymphocites; MSM: men who have sex with men; IDU: injecting drug user; MTR: multiple-tablet regimen; PI: protease inhibitors; NNRTI: non-nucleoside reverse transcriptase inhibitor; AR: adverse reaction; ARV: antiretroviral drug; ART: antiretroviral therapy

^a^ Clinical classification according to adapted criteria of Centers for Disease Control and Prevention.

^b^ Replacement of an initially prescribed active ingredient by another.

Among ART-related characteristics, antiretroviral drug switch was negatively associated with effectiveness at six and 12 months, while adherence was positively associated with effectiveness in both periods (p < 0.05). At six months, initiating treatment during hospitalization and use of MTR were associated with a lower probability of achieving effectiveness (p < 0.05), while initiating therapy in 2015 and diagnostic time above two months increased the likelihood of achieving viral suppression (p < 0.05). The extent of viral suppression at six months was strongly associated with achieving effectiveness at 12 months of treatment (OR = 7.78 and p < 0.001) (Table 3).

Tobacco use during follow-up was negatively associated with viral suppression, and illicit drug use during follow-up reduced the chance of achieving viral suppression at 12 months (p < 0.05) (Table 3).

Clinical, ART-related, behavioral and lifestyle factors remained in the final model associated with effectiveness in the multiple analysis with imputed data. Viral load above 100,000 copies/ml (p = 0.017) and presence of AIDS-defining signs and symptoms (p = 0.014) were associated with approximately 55.0% and 70.0% less likelihood of achieving viral suppression at six months, as were ARV switch (p < 0.001) and tobacco use during follow-up (p = 0.005). Only adherence to ART increased the chance of achieving viral suppression (OR = 2.11, p = 0.029) (Table 4).

**Table 4:** Multivariate analysis of effectiveness evaluated by viral suppression according to follow-up time and patients’ characteristics. Belo Horizonte, State of Minas Gerais, 2015. (n = 440)

Variable	6 months^a,b^			12 months^c,d^		
	
	OR	95%CI	p	OR	95%CI	P
Clinical						
HIV risk (MSM *versus* other groups)	-	-	-	2.44	1.04–5.69	0.040
Viral load before ART (> 100,000 copies/ml)	0.40	0.19–0.84	0.017	-	-	-
AIDS (yes)	0.47	0.26–0.86	0.014	-	-	-
ART-related						
Time between diagnosis and ART (> 2 months)	-	-	-	0.40	0.19–0.84	0.017
ARV switch^e^ (yes)	0.31	0.17–0.56	0.000	-	-	-
Adherence to ART in 6 months (yes)	2.11	1.08–4.13	0.029	-	-	-
Adherence to ART in 12 months (yes)	-	-	-	2.34	1.14–4.79	0.020
Effectiveness at 6 months (yes)	-	-	-	8.96	3.98–20.17	0.000
Behavioral						
Recent tobacco use (yes)	0.45	0.26–0.79	0.005	-	-	-
Drug use during follow-up (yes)	-	-	-	0.34	0.15–0.79	0.012

ARV: antiretroviral drug; MSM: men who have sex with men; ART: antiretroviral therapy; ROC: Receiver Operating Characteristic

^a^ Area under ROC curve = 0.785.

^b^ Pearson coeficiente correlation R^2^ = 0.99.

^c^ Area under ROC curve = 0.974.

^d^ Pearson coeficiente correlation R^2^ = 0.83.

^e^ Replacement of an initially prescribed active ingredient by another.

The multiple model with imputed data showed that achieving viral suppression at six months was the main predictor of effectiveness at 12 months (OR = 8.96, p < 0.001). In addition, adherence to ART at 12 months and belonging to the MSM category increased the likelihood of achieving viral suppression (p < 0.05), while use of illicit drugs during follow-up reduced that likelihood by 66.0% (Table 4).

## DISCUSSION

In this Brazilian cohort study, which included only ART-naïve patients, the overall effectiveness of antiretroviral therapy was high, 74.6% at six months and 83.2% at 12 months of treatment, similar to rates in developed countries^7^ ^,^ ^8^. The single-tablet regimen with tenofovir, lamivudine and efavirenz was associated with greater viral suppression at six months of treatment compared to multiple-tablet regimens.

The characteristics of the patients included in this study are similar to the profile of PLHIV in Brazil, as published in epidemiological bulletins and other national studies^3^ ^,^ ^4^ ^,^ ^a^. Patients were predominantly male, sexually infected and heterosexual. Most patients initiated ART with two NRTI associated with one NNRTI, in accordance with the Clinical Protocol and Therapeutic Guidelines in force at the time^c^.

Overall effectiveness at six months agrees with the results obtained by Cardoso et al.^4^ in a cohort of patients in Rio de Janeiro (76.9%). At 12 months of follow-up, the result (83.2%) was slightly higher than those reported in Brazilian studies carried out between 2000 and 2010 (76.1% and 77.4%)^3^ ^,^ ^4^, and higher than those observed in previous studies, 46.9% and 48.4% between 1997 and 2004^9^ ^,^ ^10^. This difference may reflect the shorter interval between diagnosis and initial therapy, according to changes in initial therapy^7^ ^,^ ^c^. In addition, the better performance of newer drugs and formulations, which reduce the occurrence of adverse events, provide greater dosage convenience, and require a lower adherence rate to be effective may have contributed to this difference^4^ ^,^ ^11^.

The simplified therapy regimen with daily ingestion of tablets only once a day is associated with the higher level of adherence of patients to ART and higher levels of viral suppression when using STR or absence of difference between groups^2^. In this cohort, effectiveness results for patients using STR were similar to results published in randomized controlled trials in ART-naïve patients, which reported viral suppression rates between 80% and 88%^12–14^, albeit with different regimen compositions.

These results reinforce the strategy of supplying generic drugs in STR in Brazil. Although the difference between groups was not statistically significant at 12 months of follow-up, there was a trend of better results for patients using this drug regarding the incidence of adverse events, switch of therapy regimens, adherence to treatment and immunological recovery, as well as higher viral suppression at six months of ART.

The effectiveness of antiretroviral therapy was influenced by clinical, behavioral and ART-related factors. Among the factors that predicted effectiveness at six months, initiating treatment with high viral load and AIDS-defining signs and symptoms were negatively associated with viral suppression, as reported in previous studies^8,15–17^. Initiating ART regardless of CD4+ cell counts has been adopted in Brazil since 2013. Recent studies indicate a 50% reduction in the incidence of serious AIDS-related events, such as death and opportunistic diseases, among patients who initiate ART early (CD4+ count > 500 cells/mm^3^)^18^ ^,^ ^19^. Patients who initiated ART within a shorter interval after diagnosis had a greater chance of achieving viral suppression at 12 months in this study.

Despite knowledge of these benefits and policies introduced to increase access to diagnosis and treatment, such as expansion of testing sites and availability of quick diagnostic tests^h^, most patients initiated ART with advanced immunosuppression. The same pattern was observed in Brazilian studies in previous years^3^ ^,^ ^20^ and may be related to difficult access to healthcare services and lack of knowledge and awareness of the population about HIV risks. This results in late search for healthcare services and, consequently, late initiation of ART^20^.

The MSM were more likely to achieve viral suppression after 12 months of treatment compared to other risk or exposure categories. This result may reflect a greater involvement of those patients in continuous healthcare^21^. However, further studies are needed for inferences in this population.

The ARV switch and ART adherence influenced viral suppression. The ARV switch is associated with lower adherence to treatment and may be more closely related to the occurrence of adverse events and intolerance rather than virologic failure^22^ ^,^ ^23^. It may also be due to timely monitoring of effectiveness.

The relationship between adherence to treatment and ART effectiveness is well documented in the literature^8^ ^,^ ^10^ ^,^ ^16^ and the lowest level of adherence required to ensure the effectiveness of antiretroviral drugs is between 80.0% and 95.0%^11^. In this study, non-adherence was observed in the medical records of 16.6% of patients over six months and 26.6% over 12 months. Such data are worrying, since medical records underestimate actual non-adherence figures. Non-adherence may lead to the development of viral resistance, progression of the disease, increased morbidity and mortality due to AIDS and contribute to increase patient care costs^10^ ^,^ ^11^.

Viral suppression at six months was the main predictor of effectiveness in one year^16^ ^,^ ^17^. No patients in this study had previously used ART. Considering the prevalence of HIV-resistant strains in Brazil (11.6%)^24^, failure of viral suppression is probably due to non-adherence, although resistance to NNRTI antiretroviral drugs has increased in Minas Gerais^25^. Checking patients’ adherence to therapy and carrying out actions to increase it are as important as timely monitoring of viral load and performance of genotyping tests to ensure adequate response to treatment.

Use of substances such as tobacco and illicit drugs during follow-up were associated with lower likelihood of achieving viral suppression. Tobacco use among PLHIV has been linked to worse clinical outcomes, such as increased viral load, reduced CD4+ cell count and increased occurrence of opportunistic infections^26^ ^,^ ^27^. The negative impact of tobacco use and its high prevalence in this population evidence the need to enhance non-smoking programs in HIV/AIDS specialized units.

The use of illicit substances among PLHIV may be related both to the actual source of infection and to diagnosis coping mechanisms^28^. The use of these substances is related to lower levels of adherence to treatment, lower viral suppression and lower levels of CD4+^28^ ^,^ ^29^. Worse results are also reported with previous use of illicit drugs^3^ ^,^ ^29^ ^,^ ^30^. Treating chemical dependency, introducing other methods to help cope with the diagnosis and raising the health team’s awareness of the problem^28–30^ are possible strategies to improve the clinical results of these patients.

This study has limitations, such as poor accuracy of measurements collected from medical records and high percentage of missing data. The strategies used to minimize their effect were the elaboration of clinical scenarios and data imputation, with no change in the trend of results. Study strengths include the high quality of collection, the broad inclusion of confounding factors, and the robustness of the final model in both follow-up periods

The incidence of viral suppression at six and 12 months in patients with no prior use of ART was high, with differences between patients using STR and MTR. Clinical, behavioral, lifestyle, and ART-related factors influenced viral suppression. They also demonstrated the need for interventions to improve diagnosis and the timely initiation of treatment, patients’ adherence to therapy, and the reduction of tobacco and illicit drugs use, so as to optimize treatment outcome and contribute to quality of life and reduction of HIV transmission.

## Footnotes

a Boletim Epidemiológico de Aids e DST. Brasília (DF): Ministério da Saúde, Secretaria de Vigilância em Saúde; 2016 [cited 2017 Mar 3];5(1). Available from: http://www.aids.gov.br/pt-br/pub/2016/boletim-epidemiologico-de-aids-2016

b Ministério da Saúde (BR), Secretaria de Vigilância em Saúde, Departamento de Vigilância, Prevenção e Controle das Infecções Sexualmente Transmissíveis, do HIV/AIDS e das Hepatites Virais. Relatório de monitoramento clínico do HIV. Brasília (DF); 2017. Available from: http://www.aids.gov.br/pt-br/pub/2017/relatorio-de-monitoramento-clinico-do-hiv

c Ministério da Saúde (BR), Secretaria de Vigilância em Saúde, Departamento de Vigilância, Prevenção e Controle das Infecções Sexualmente Transmissíveis, do HIV/AIDS e das Hepatites Virais. Protocolo clínico e diretrizes terapêuticas para manejo da infecção pelo HIV em adultos. Brasília (DF); 2013.

d World Health Organization. Consolidated guidelines on the use of antiretroviral drugs for treating and preventing HIV infection: recommendations for a public health approach. 2.ed. Geneva: WHO; 2016 [cited 2017 Apr 20]. Available from: http://www.who.int/hiv/pub/arv/arv-2016/en/

e Ministério da Transparência e Controladoria-Geral da União (BR). Portal da Transparência: gastos diretos do Governo por ação: exercício de 2016. Brasília (DF); 2017 [cited 2017 Mar 3]. Available from: http://www.transparencia.gov.br/PortalComprasDiretas

f Ministério da Saúde (BR), Secretaria de Vigilância em Saúde, Programa Nacional de DST e Aids. Critérios de definição de casos de AIDS em adultos e crianças. Brasília (DF); 2004 [cited 2017 Apr 20]. Available from: http://bvsms.saude.gov.br/bvs/publicacoes/criterios_definicao_AIDS_adultos_criancas.pdf

g World Health Organization. ICD-10: international statistical classification of diseases and related health problems: tenth revision. 2.ed. Geneva: WHO; 2004 [cited 2017 Apr 20]. Available from: http://apps.who.int/iris/handle/10665/42980

h Ministério da Saúde (BR), Secretaria de Vigilância em Saúde, Departamento de Vigilância, Prevenção e Controle das IST, do HIV/AIDS e das Hepatites Virais. Relatório de monitoramento clínico do HIV - 2016. Brasília (DF); 2016 [cited 2017 Apr 20]. Available from: http://www.aids.gov.br/pt-br/pub/2016/relatorio-de-monitoramento-clinico-do-hiv-2016
